# Cardiac Dysfunction Induced by Obesity Is Not Related to *β*-Adrenergic System Impairment at the Receptor-Signalling Pathway

**DOI:** 10.1371/journal.pone.0138605

**Published:** 2015-09-21

**Authors:** Artur Junio Togneri Ferron, Bruno Barcellos Jacobsen, Paula Grippa Sant’Ana, Dijon Henrique Salomé de Campos, Loreta Casquel de Tomasi, Renata de Azevedo Mello Luvizotto, Antonio Carlos Cicogna, André Soares Leopoldo, Ana Paula Lima-Leopoldo

**Affiliations:** 1 Center of Physical Education and Sports, Department of Sports, Federal University of Espírito Santo, Vitória, Espírito Santo, Brazil; 2 Department of Clinical and Cardiology, School of Medicine, UNESP- Univ. Estadual Paulista, Botucatu, São Paulo, Brazil; 3 Instituto de Ciências da Saúde, Federal University of Mato Grosso, Sinop, Mato Grosso, Brazil; Temple University, UNITED STATES

## Abstract

Obesity has been shown to impair myocardial performance. Some factors have been suggested as responsible for possible cardiac abnormalities in models of obesity, among them beta-adrenergic (βA) system, an important mechanism of regulation of myocardial contraction and relaxation. The objective of present study was to evaluate the involvement of βA system components in myocardial dysfunction induced by obesity. Thirty-day-old male *Wistar* rats were distributed in control (C, n = 25) and obese (Ob, n = 25) groups. The C group was fed a standard diet and Ob group was fed four unsaturated high-fat diets for 15 weeks. Cardiac function was evaluated by isolated papillary muscle preparation and βA system evaluated by using cumulative concentrations of isoproterenol and Western blot. After 15 weeks, the Ob rats developed higher adiposity index than C rats and several comorbidities; however, were not associated with changes in systolic blood pressure. Obesity caused structural changes and the myocardial responsiveness to post-rest contraction stimulus and increased extracellular calcium (Ca^2+^) was compromised. There were no changes in cardiac function between groups after βA stimulation. The obesity was not accompanied by changes in protein expression of G protein subunit alpha (Gs*α*) and βA receptors (β_1_AR and β_2_AR). In conclusion, the myocardial dysfunction caused by unsaturated high-fat diet-induced obesity, after 15 weeks, is not related to βAR system impairment at the receptor-signalling pathway.

## Introduction

Obesity is a complex disease characterised by excessive accumulation of adipose tissue that affects 30% of the world population and 10.5 million Brazilians [1,2]. Obesity is a consolidated nutritional problem associated with insulin resistance, type 2 diabetes mellitus, dyslipidaemia, some types of cancer and cardiovascular diseases [3,4].

Clinical research shows that excess fat causes cardiac abnormalities such as haemodynamic, morphologic and functional changes that correlate with the duration and intensity of obesity [5,6]. Within this context, experimental obesity using genetic models [7,8] or dietary manipulations [9–12] has become an important alternative for the study of obesity and cardiac function.

Several studies have shown that obesity induced by different types of high-fat diets and/or highly energetic diets promotes myocardial dysfunction in rodents [10,13,14]. In contrast, others authors have demonstrated that high-fat feeding was not sufficient to cause significant cardiac abnormalities [9,15]. Recent researches performed in our laboratory show that obese rats fed a high-fat diet for 15 weeks presented myocardial dysfunction at the baseline condition and after inotropic manoeuvres [14,16].

Although a variety of changes and/or damage in the cardiac performance occur in both obese humans and obese rodents, the mechanisms responsible for these alterations are not well established. Several factors have been suggested as possible causes of the cardiac abnormalities in obese models [10,13]. Among these possible causes, the βA system is an important mechanism of myocardial contraction and relaxation regulation in physiological conditions and pathological situations [16–20].

The beta-adrenergic (βA) pathway is composed of βA receptors (βAR) coupled to G proteins (G_s_ and G_i_), adenylate cyclase (AC) and cyclic adenosine monophosphate (cAMP) [21,22]. The human heart contains all three βAR subtypes, β_1_AR, β_2_AR and β_3_AR [23]. β_1_AR is the predominant subtype in the normal myocardium, representing 75–80% of total βAR density, followed by β_2_AR, which comprises approximately 15–18% of the total cardiomyocyte βARs; the remaining 2–3% is β_3_ARs [24]. The principal role of βARs in the heart is the regulation of cardiac rate and contractility in response to catecholamines [24]. Both receptors β_1_AR and β_2_AR are coupled to G_s_ protein, activating adenylate cyclase and subsequently increasing the levels of cAMP. The accumulation of cAMP results in higher activation of protein kinase A (PKA), which triggers changes in intracellular calcium (Ca^2+^) [22–26]. Thus, the stimulation of β_1_ARs (mainly) and β_2_ARs (to a lesser extent) can increase cardiac contractility (positive inotropic effect), frequency (positive chronotropic effect), and rate of relaxation (lusitropic effect) [25].

Although the βAR and the G protein in cardiomyocytes play important functions in the regulation of cardiac performance, researches have shown that in pathological conditions, including diabetes and heart failure, changes in the expression and/or activity of βA components promote functional damage [27–33]. However, few studies have evaluated the βA system in experimental models of obesity [34,35]. Carroll et al. [34,35] showed that after 12 weeks of high-fat diet, obesity promotes a reduction in responsiveness of the isolated heart to isoproterenol, a non-selective βAR agonist, without alterations in β-receptor density and affinity in obese female *New Zealand* white rabbits.

Given the lack of studies evaluating the relationship between obesity with a high unsaturated fat diet and the β-adrenergic pathway in cardiac function, the purpose of this study was to investigate the role of β-adrenergic components on myocardial dysfunction induced by obesity. Our hypothesis is that functional impairment in obese rats is related to lower expression and/or activity of β-adrenergic receptors and reduced levels of the myocardial Gs_*α*_ protein.

## Material and Methods

### Animal care

Thirty-day-old male *Wistar* rats (≈ 150 g) obtained from the Animal Center of Botucatu Medical School (Botucatu, São Paulo, Brazil) were housed in individual cages. The environment was controlled in terms of light (12 h light/dark cycle starting at 6 am), clean-air room temperature (23±3°C), and relative humidity (60±5%). All experiments and procedures were performed in accordance with the Guide for the Care and Use of Laboratory Animals published by the National Research Council (1996) and approved by the Espírito Santo Medical School Ethics Committee (UFES, Espírito Santo, ES, Brazil) under number 017.

### Experimental protocol

After 7 days of acclimatization, the rats were randomly distributed into 2 groups: control (C, n = 25) and obese (Ob, n = 25). The C group was fed a standard diet (RC Focus 1765) containing 12.3% of its kcal from fat, 57.9% from carbohydrates, and 29.8% from protein. The Ob animals were fed four high-fat diets (RC Focus 2413, 2414, 2415, and 2416), only differing in their flavoring, but not different in micro or macronutrients. The high-fat diets contained 49.2% of their kcal from fat, 28.9% from carbohydrates, and 21.9% from protein as previously described [14]. The high-fat diets were calorically rich (high-fat diet = 3.65 kcal/g *versus* low-fat diet = 2.95 kcal/g) due to its higher fat energy (consisting of saturated and unsaturated fatty acids, which provided 20 and 80% of the fat-derived calories, respectively). Animals had free access to water and chow (50 g/day); after 24 hours the amount of diet that was not consumed was measured. At week 3 of this study, the beginning of obesity based on body weight gain was established, which was previously determined by our group [36]. At this time-point, the C and Ob rats were maintained on their respective diets for an additional 15 consecutive weeks.

After starting the experimental protocol, food consumption (FC), calorie intake (CI), feed efficiency (FE), and body weight (BW) were recorded weekly. CI was calculated as follows: CI = average weekly food consumption calorie value of each diet. FE (%) is the ability to convert calorie intake to BW and was determined as the mean BW gain (g)/total calorie intake (kcal) x100 [37].

### Determination of obesity

A criterion based on the adiposity index was used to determine obesity according to several authors [9,38,39]. After 15 weeks of developing obesity, animals were anaesthetised by ketamine injection (50 mg/kg) and xylazine (0.5 mg/kg), decapitated, and thoracotomised, and the fat pads of adipose tissue were dissected and weighed. The adiposity index was calculated using the following formula: adiposity index = (total body fat (BF)/final body weight) x 100. BF was measured from the sum of the individual fat pad weights: BF = epididymal fat + retroperitoneal fat + visceral fat.

### Characterisation of groups

After 15 weeks of experimental protocol, a 95% confidence interval (CI) was built for the adiposity index from the Ob and C rats and was adopted as the separation point (SP) between the groups, the midpoint between the upper limit and the lower limit C of the Ob. From this point, the control animals with an adiposity index above of SP and the Ob animals with an adiposity index below the SP were excluded from the C and Ob groups, respectively, ensuring the homogeneity of the treated and control groups. This criterion was adopted because biological experimentation can occur misclassification, in other words, animals submitted to high-fat diet should be classified as obese and can exhibit characteristics of control animals. Therefore, animals submitted to different diet models do not always present the expected response. This fact can lead to erroneous animal classification and, consequently, false conclusions.

### Comorbidities and hormones associated with obesity

Because the rat models of diet-induced obesity may develop some of characteristics of human obesity, such as hypertension, glucose (GL) intolerance, insulin resistance, dyslipidaemia, hyperinsulinemia, and hyperleptinemia, these were evaluated in all groups.

### Systolic blood pressure (SBP)

SBP evaluation was assessed in conscious rats by the non-invasive tail-cuff method with a NarcoBioSystems® Electro-Sphygmomanometer (International Biomedical, Austin, TX, USA). The animals were warmed in a wooden box (50 x 40 cm) between 38–40°C with heat generated by two incandescent lamps for 4–5 minutes to cause vasodilation artery tail and were then transferred to an iron cylindrical support that was specially designed to allow total exposure of the animal's tail [40]. After this procedure, a cuff with a pneumatic pulse sensor was attached to the tail of each animal. The cuff was inflated to 200 mmHg pressure and subsequently deflated. The blood pressure values were recorded on a Gould RS 3200 polygraph (Gould Instrumental Valley View, Ohio, USA). The average of two pressure readings was recorded for each animal.

### Glucose (GL) tolerance

Following 15 weeks of treatment, GL tolerance was evaluated by glucose tolerance test. Experiments were performed on all rats (C and Ob groups), and the animals were deprived of food for 4–6 h [41]. After fasting, a blood sample was collected from the tip of the tail in a heparinised tube. The basal blood GL level of each animal was immediately determined using a handheld glucometer (Accuchek Advantage; Roche Diagnostics Co., Indianapolis, IN). Subsequently, an injection of glucose solution (2 g/kg body weight) dissolved in water was administered intraperitoneally (Sigma-Aldrich®, St Louis, MO, USA), and the blood GL levels were measured after 15, 30, 60, 90, and 120 minutes [42].

### Homeostatic model assessment index (HOMA-IR)

HOMA-IR was expressed as an index of insulin resistance and calculated using the following formula: HOMA-IR = [fasting GL (mmol/L) X fasting insulin (mU/mL)]/22.5 [43]. All rats ate normally and regained their body weights within 1 day after this regimen.

### Cholesterol, triglycerides, hyperinsulinemia, and hyperleptinemia

At the end of the experimental period, the animals were fasted for 12–15 h, anaesthetised with an intraperitoneal injection of ketamine (50 mg/kg) and xylazine (0.5 mg/kg), and euthanised by decapitation. Blood samples were collected in heparinised tubes, and the serum was separated by centrifugation at 3000 × g for 15 minutes at 4°C and stored at -80°C until further analysis. Serum was analysed for levels of triglycerides (TG), total cholesterol (T-Chol), high-density lipoprotein cholesterol (HDL), low-density lipoprotein cholesterol (LDL), and hormones (insulin and leptin). TG, T-Chol, HDL and LDL were measured with an automatic enzymatic analyser system (Biochemical analyser BS-200, Mindray, China). Leptin and insulin levels were determined with the enzyme-linked immunosorbent assay (ELISA) method using commercial kits (Linco Research Inc., St. Louis, MO, USA).

### Post-death morphological analysis

Rats were euthanised by thoracotomy, and the hearts, ventricles and tibia were separated, dissected, weighed and measured. Cardiac remodelling at the macroscopic level, which identifies the presence or absence of cardiac hypertrophy, was determined by analysing the following parameters: heart weight (HW), left ventricle (LV) weights, HW and LV/tibia length ratios.

### Myocardial function

Myocardial function was evaluated by studying isolated papillary muscles from the LV. This procedure has been utilised by various authors [14,16,44]. This preparation permitted the measurement of the capacity of cardiac muscle to shorten and develop forces independent of influences that can modify *in vivo* mechanical performance of the myocardium, such as the heart rate, preload, and afterload. Briefly, at the time of investigation, rats were anaesthetised with an intraperitoneal injection of ketamine (50 mg/kg) and xylazine (0.5 mg/kg) and euthanised by decapitation. The hearts were quickly removed and placed in oxygenated Krebs-Henseleit solution at 28°C. The LV papillary muscles from the C (n = 20) and Ob rats (n = 17) were dissected, mounted between two spring clips, placed vertically in a chamber containing Krebs-Henseleit solution (118.5 mM NaCl; 4.69 mM KCl; 2.5 mM CaCl_2_; 1.16 mM MgSO4; 1.18 mM KH_2_PO4; 5.50 mM GL, and 24.88 mM NaHCO_3_) and maintained at 28°C with a thermostatic water circulator. The bathing solution was bubbled with 95% oxygen and 5% carbon dioxide, with a pH of 7.4. The lower spring clip was attached to a 120T-20B-force transducer (Kyowa, Tokyo, Japan) by a thin steel wire (1/15,000 inch), which passed through the mercury seal at the bottom of the chamber. The upper spring clip was connected with a thin steel wire to a rigid lever arm, above which a micrometer stop was mounted for adjusting the muscle length. The muscle preparation was placed between two parallel platinum electrodes (Grass E8, GRASS Technologies, An Astro-Med, Inc. Product Group, West Warwick, RI, USA) and stimulated at a frequency of 0.2 Hz (12 pulses/min) with 5 ms square-wave pulses. Voltage was set to a value 10% greater than the minimum required to produce a maximal mechanical response.

The muscles were contracted isotonically with light loads for 60 min, loaded (50 g) to contract isometrically and stretched to the maximum of their length-tension curves. After a 5-min period during which preparations underwent isotonic contractions, muscles were again placed under isometric conditions, and the peak of the length-tension curve (*Lmax*) was carefully determined. A 15-min period of stable isometric contraction was imposed prior to the experimental period, during which one isometric contraction was then recorded. Conventional mechanical parameters at *Lmax* were calculated from isometric contraction: maximum developed tension normalised per cross-sectional area (DT [g/mm^2^]), resting tension normalised per cross-sectional area (RT [g/mm^2^]), positive (+dT/dt [g/mm^2^/s]) and negative (-dT/dt [g/mm^2^/s]) tension derivative normalised per cross-sectional area of papillary muscle (CSA).

### Inotropic and lusitropic manoeuvres

To determine the mechanism by which obesity induces negative inotropic effects on myocardial function, the papillary muscles were evaluated under the baseline condition of 2.5 mM Ca^2+^ and after inotropic manoeuvres, namely increases in extracellular Ca^2+^ concentration (to test the effect on the myofilament machinery) and post-rest contraction (PRC), mainly related to sarcoplasmic reticulum (SR) storage and release capacity [45,46].

Inotropic responses were recorded at 5 minutes after the addition of each dose of extracellular Ca^2+^ (0.5, 1.0, 1.5, 2.0, and 2.5 mM) to the bathing solution. PRC was studied at an extracellular Ca^2+^ concentration of 0.5 mM, where the stimulus was interrupted for 10, 30, 60 and 90 seconds before restarting the stimulation. During resting conditions, in the rat myocardium, Ca^2+^ accumulates in the SR above and beyond what accumulated during the regular stimulation, and the first beat after the rest interval (B1) is stronger than the beat immediately prior to the rest interval (B0) [47]. All mechanical values of manoeuvres were expressed as the mean percent of baseline data and were calculated as follows: D = (M2-M1)/M1x100, where M1 was the value in the baseline condition and M2 was the value after the inotropic and lusinotropic manoeuvres.

### Analysis of β-adrenergic system

The evaluation of the β-adrenergic system was performed with contractile responsiveness in the papillary muscle to isoproterenol and by protein expression of myocardial adrenergic receptors β_1_AR, β_2_AR and G_s_ *α*. The β-adrenoceptor system was studied to test the integrity of the beta-adrenergic complex system, sensitivity to troponin-C, and the calcium uptake by SR.

### β-adrenergic receptor (βAR) responsiveness

β-adrenergic receptors (βAR) are important regulators of normal and pathologic cardiac function and are expressed in cardiomyocytes [48]. βAR receptor activity was evaluated by determining the dose-response relationship between the isoproterenol and conventional mechanical parameters of papillary muscle at *Lmax*. After baseline measurements had been determined, isoproterenol was added to the bath in the presence of 1.0 mM [Ca^2+^] to yield progressively increasing concentrations of 10^−7^, 10^−6^ and 10^−5^ mol/L. Contractile response stabilised approximately 3 to 5 minutes after adding each isoproterenol dose. Data were then sampled and expressed as the mean percent of stimulation (%).

At the end of the study, the parameters used to characterize the papillary muscle were length (mm), weight (mg), and CSA (mm^2^). The CSA was calculated from the length and weight of papillary muscle, assuming uniformity and a specific gravity of 1.0. The muscle length at *Lmax* was measured with a cathetometer (Gartner Scientific Corporation, Chicago, IL, USA), and the muscle between the two clips was blotted dry and weighed.

### Protein expression of βAR and G_s_α

The myocardial levels of β_1_AR, β_2_AR and G_s_ *α* in both groups were evaluated with Western blot. Briefly, LV samples were frozen in liquid nitrogen from C (n = 7) and Ob (n = 7) rats and homogenised in a buffer containing 50 mM potassium phosphate buffer (pH 7.0), 0.3 M sucrose, 0.5 mM dithiothreitol (DTT), 1 mM ethylenediaminetetraacetic acid (EDTA) (pH 8.0), 0.3 mM phenylmethylsulfonyl fluoride (PMSF), 10 mM sodium fluoride (NaF), and phosphatase inhibitor cocktail (1:100; Sigma-Aldrich). The samples were subjected to sodium dodecyl sulfate/polyacrylamide gel electrophoresis (SDS-PAGE) in 8–12% polyacrylamide gels depending on the molecular weight of the protein. After electrophoresis, the proteins were electrotransferred to nitrocellulose membranes (Bio-Rad Biosciences; NJ, USA). Equal loading of the samples (50 μg) and transfer efficiencies were monitored with 0.5% Ponceau S staining of the membrane. The blotted membrane was blocked (5% non-fat dry milk, 10 mM Tris-HCl (pH 7.6), 150 mM NaCl, and 0.1% Tween 20) for 2 h at room temperature and then incubated overnight at 4°C with specific antibodies against β_1_AR (Abcam, Cambridge, MA, USA; ab3442, 1:1000), β_2_AR (Abcam, Cambridge, MA, USA; ab36956, 1:1000) and G_s_α (Abcam, Cambridge, MA, USA; ab97663, 1:500). Binding of the primary antibody was detected with peroxidase-conjugated secondary antibodies (rabbit or mouse, depending on the protein, for 2 h at room temperature), developed using enhanced chemiluminescence (Amersham Biosciences, NJ, USA), and detected with autoradiography. β-actin was used as an internal control (Santa Cruz Biotechnology, CA, USA; SC81178, 1:1000). The blots were developed using an enhanced chemiluminescent (ECL) Super Signal® West Pico Chemiluminescent Substrate (Thermo Scientific, Rockford, IL, USA) and analysed by using a densitometer (GS-710 calibrated imaging densitometer, Bio-Rad lab, CA, USA).

### Statistical analysis

Data from general characteristics, comorbidities, myocardial function and western blot analysis were reported as the means ± standard deviation (SD). Comparisons between the groups were performed using Student's t test for independent samples. Repeated-measures two-way analysis of variance (ANOVA) was used to evaluate the positive and negative inotropic effects on myocardial function and βAR responsiveness to isoproterenol. When significant differences were found (p<0.05), Student-Newman-Keuls post-hoc test for multiple comparisons was carried out. The level of significance considered was 5% (p<0.05).

## Results

**Fig 1:**
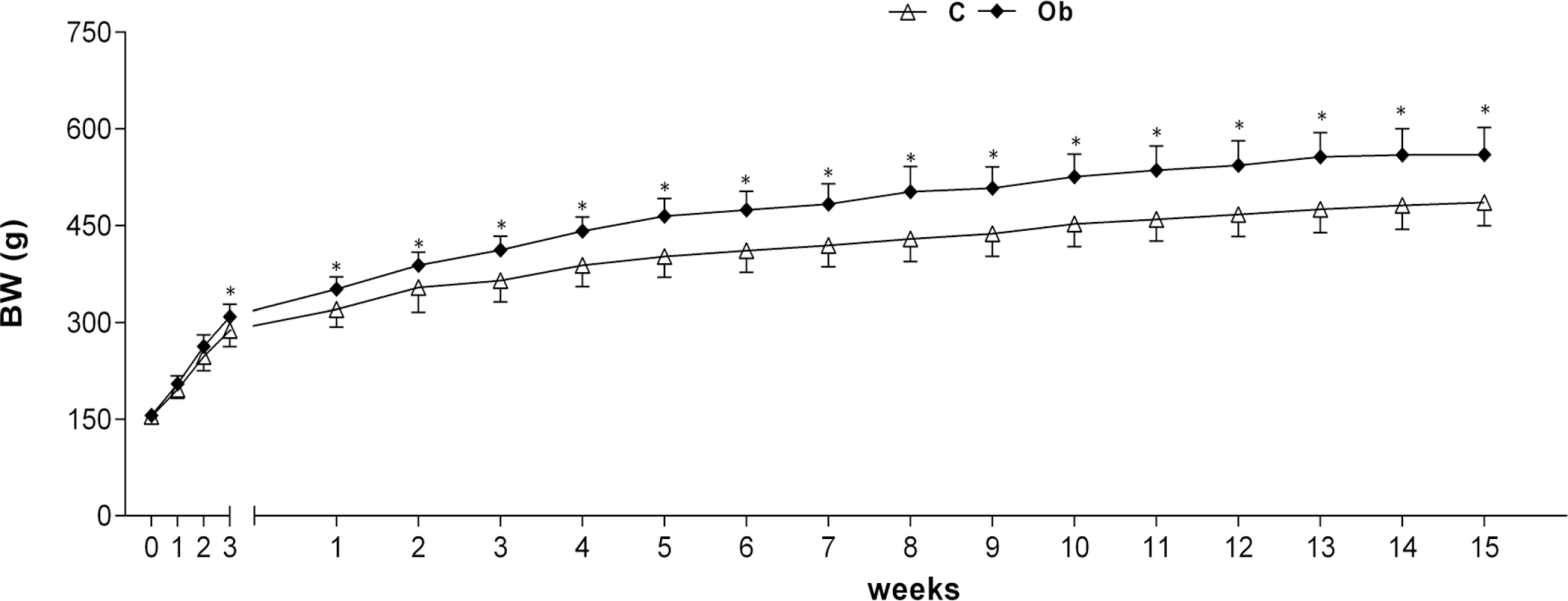
Changes in body weight (BW) during 15 weeks of experimental protocol after the initiation of obesity at week 3. Data presented as the mean ± SD. * p<0.05 *versus* C. Two way ANOVA for independent samples and Bonferroni’s post-hoc analysis.

From the exclusion criteria mentioned in the characterisation of the groups, twenty rats remained in the study in the C group (C, n = 20) and seventeen remained in the obese group (Ob, n = 17). The change in weekly weights of the groups was similar in the first two weeks of treatment; after the 3rd week, the body weights of the Ob rats were significantly higher than those of the C rats (Fig 1). This time was associated with characterization of initial moment of obesity. After determining the initial moment of obesity, the weight of the Ob animals remained significantly higher than that of the C animals during the 15 weeks of the experiment.

**Fig 2:**
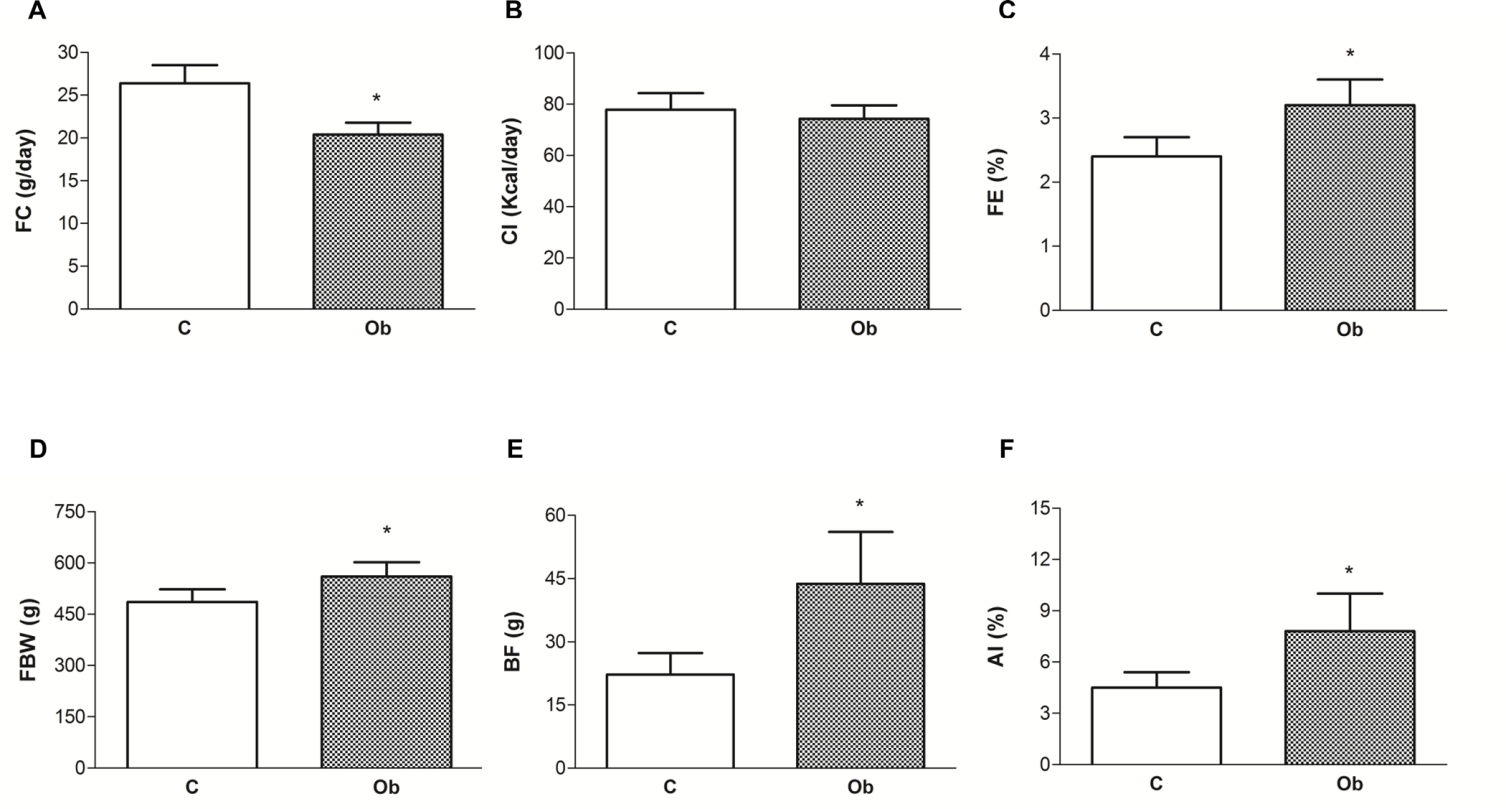
General characteristics of rats after 15 weeks of treatment. Control (C; n = 20) and obese (Ob; n = 17) groups. n = number of animals; (A) FC—food consumption; (B) CI—calorie intake; (C) FE—feed efficiency; (D) FBW—final body weight; (E) BF—body fat; (F) AI—adiposity index. Data presented as the means ± standard deviation. *p<0.05 *versus* C. Student’s t-test for independent samples.

The general characteristics of the animals are shown in Fig 2A–2F. The high-fat diet promoted a substantial elevation of body fat and adiposity index in the Ob rats compared to those of the C rats. Specifically, the Ob rats had a significantly higher final body weight (15.2%), body fat content (97.3%) and adiposity index (73.3%) when compared to the C rats, respectively (Fig 2D–2F). Despite the greater amount of energy from high-fat diets, the calorie intake was similar between the groups due to the reduced FC in the Ob rats compared to the C rats (Fig 2A and 2B). In addition, feed efficiency was higher in the Ob group (33.3%) than in the C group (Fig 2C).

**Fig 3:**
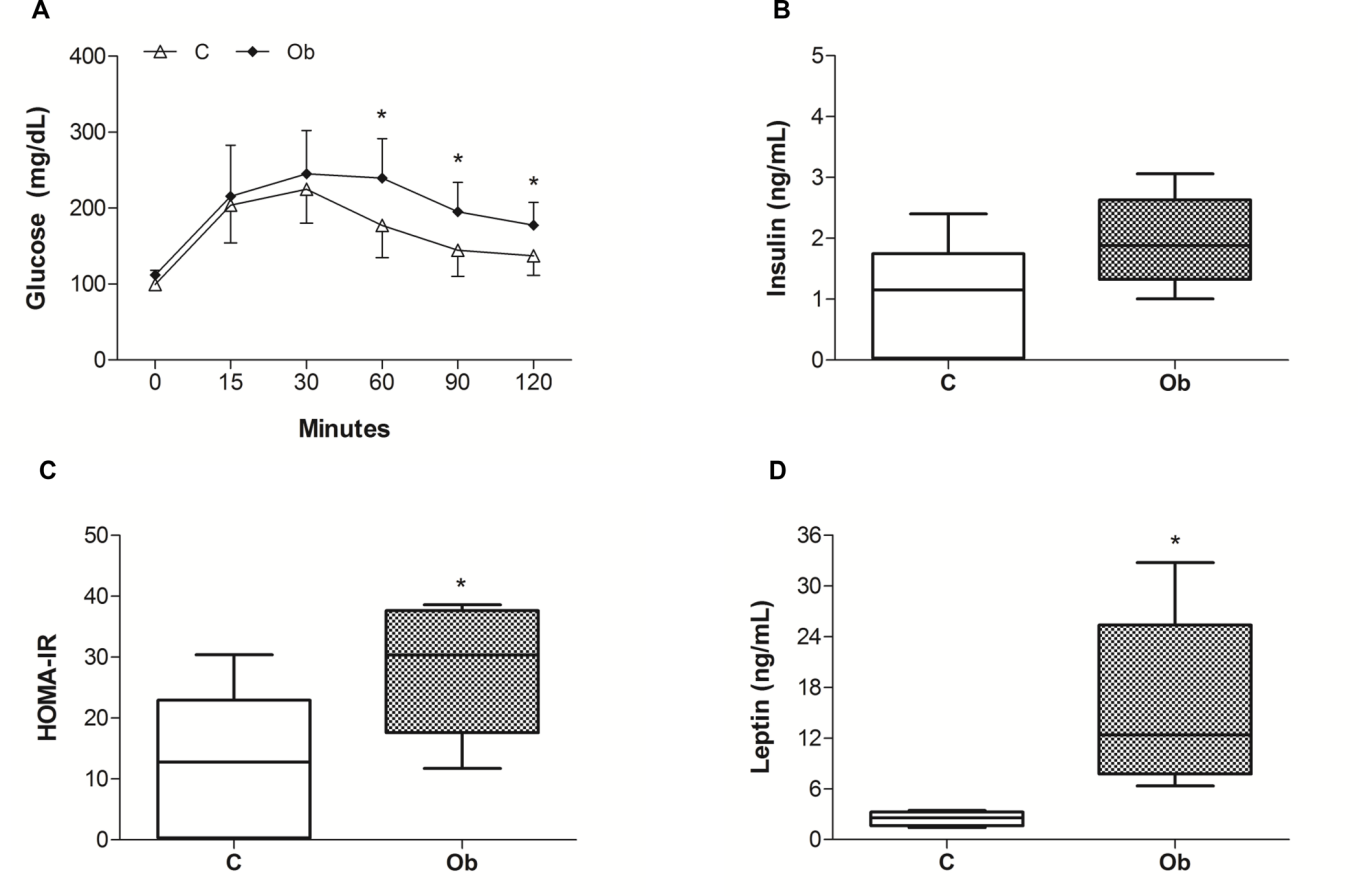
(A) Glucose tolerance profile, (B and D) hormone serum levels and (C) homeostatic model assessment index (HOMA-IR). Control (C; n = 20) and obese (Ob; n = 17) groups. n = number of animals. (A) Data presented as the means ± standard deviation; Two way ANOVA for independent samples and Bonferroni’s *post-hoc* analysis. (B, C and D) Values shown are median ± interquartile range; Mann-Whitney test. *p<0.05 *versus* C.

The comorbidities, hormones and cardiac morphology associated with obesity are summarised in Table 1 and Fig 3A–3D. There were no significant differences in the systolic blood pressure, T-Chol, HDL and LDL between the groups. In addition, the glucose tolerance profile and HOMA-IR were significantly affected by exposure to obesity (Ob > C, p< 0.05). Although there was no difference in glucose levels under baseline condition and after 15 and 30 minutes, Ob rats presented higher levels of glucose at 60, 90 and 120 minutes than the C rats (Fig 3A). Moreover, the insulin and HOMA-IR were higher in the Ob rats than in the C rats (Fig 3B and 3C). These findings reveal compromised GL tolerance and insulin resistance in the Ob rats (Fig 3A–3C). Furthermore, TG and leptin levels were higher in the Ob rats than in the C rats (Table 1 and Fig 3D, respectively). The cardiac morphological profile rats are displayed in Table 1. Absolute heart and LV weights and these values in relation to tibia length were significantly elevated in the Ob compared to the C group (Table 1).

**Table 1:** Systolic blood pressure, lipid profile and cardiac morphology

	Groups	
Variables	C	Ob
**SBP (mmHg)**	126 ± 11	131 ± 12
**TG (mg/dL)** ^#^	44.5 ± 15.6	69.9 ± 61.5*
**T-Chol (mg/dL)**	62.4 ± 11.5	67.5 ± 18.0
**HDL (mg/dL)**	23.5 ± 3.0	26.6 ± 5.8
**LDL (mg/dL)**	31.3 ± 5.7	36.5 ± 9.4
**HW (g)**	1.17 ± 0.09	1.31 ± 0.13*
**LVW (g)**	0.86 ± 0.06	0.94 ± 0.08*
**HW/Tibia length (g/cm)**	0.27 ± 0.02	0.30 ± 0.03*
**LVW/Tibia length (g/cm)**	0.20 ± 0.01	0.21 ± 0.02*

Data presented as means ± SD. control (C) and obese (Ob) groups; n: animals numbers; Systolic blood pressure and lipid profile (*n* = 7 animals); SBP: systolic blood pressure; TG: triglycerides; T-Chol: total cholesterol; HDL: high-density lipoprotein; LDL: Low-density lipoprotein. Cardiac parameters *(C*, *n = 20; Ob*, *n = 17)*; HW: heart weight; LVW: left ventricle weight;

*p<0.05 versus C; Student’s t-test for independent samples.

**Fig 4:**
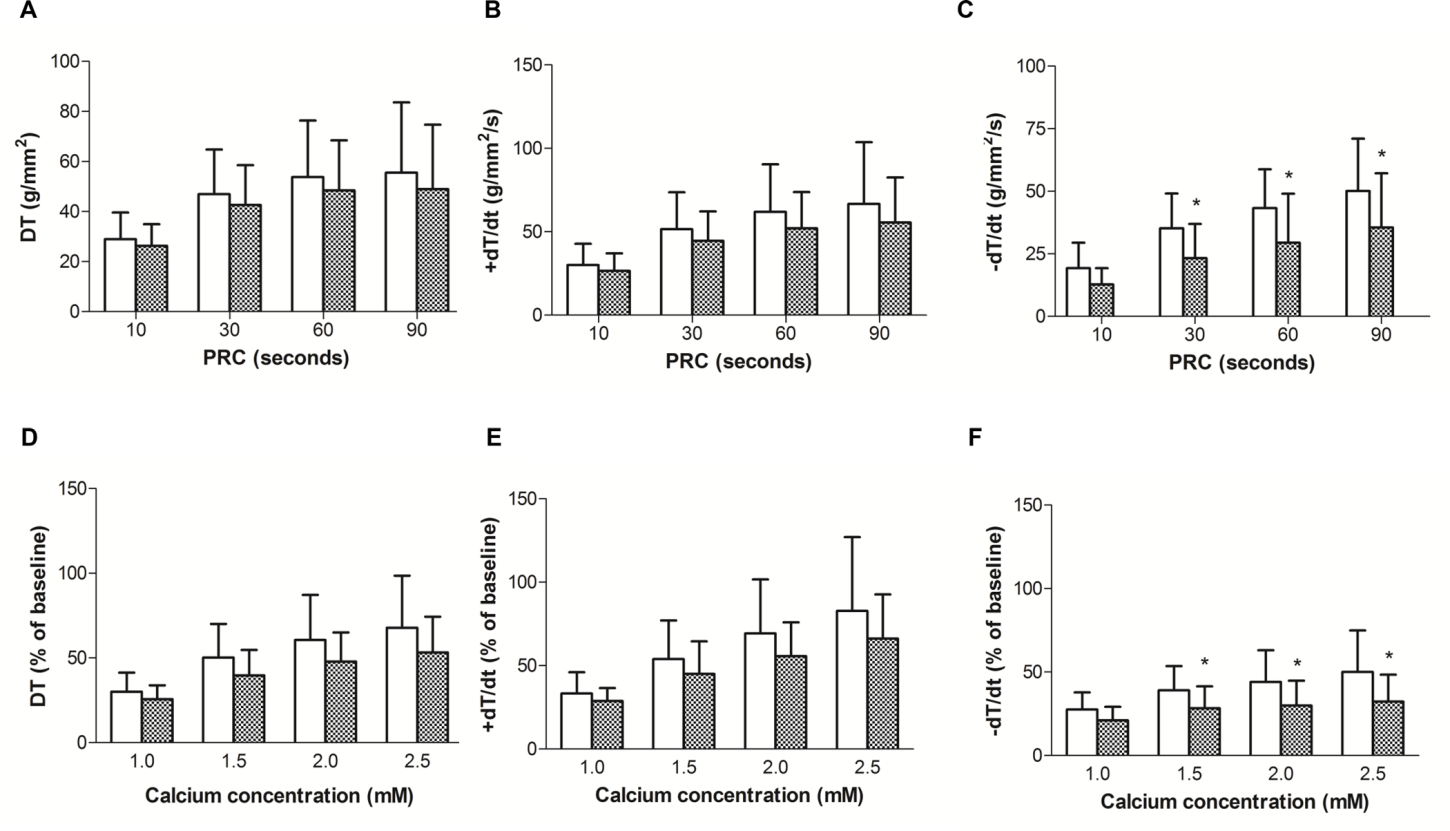
(A, B and C) Post-rest contraction (PRC) and (D, E and F) effects of increasing extracellular Ca^2+^ concentration in papillary muscles from control (white bars) and obese rats (cross-hatched bars). Baseline calcium concentration (0.5 mM) is presented as 100%. Maximum developed tension normalised per cross-sectional area [DT, g/mm^2^] and positive [+dT/dt, g/mm^2^/s] and negative [-dT/dt, g/mm^2^/s] tension derivative normalized per cross-sectional area of the papillary muscle. Data presented as the mean percent of baseline ± standard deviation.*p < 0.05 *versus* C. Repeated-measures two-way ANOVA and Student-Newman-Keuls *post-hoc t*est.

The analyses of myocardial papillary muscle function obtained at baseline condition with Ca^2+^ concentration of 2.5 mM are shown in Table 2. Obesity did not cause functional impairment because the parameters analysed (DT, RT, +dT/dt and-dT/dt) were similar between the groups. In addition, the papillary muscle CSA showed no difference between the C and Ob rats. PRC and the effects of increasing extracellular Ca^2+^ concentration in the isolated papillary muscle function are shown in Fig 4A–4F. The results shown in Fig 4C indicate that PRC induced a greater response in -dT/dt in the C rats than in the Ob rats. The -dT/dt was significantly diminished in the obese myocardium after 30, 60 and 90 s of stimulus cessation. The mean percent of -dT/dt was 23.25 ± 13.70%, 29.46 ± 19.58%, 35.50 ± 21.71% in the baseline Ob group *vs*. 35.17 ± 13.94%, 43.26 ± 15.57%, 50.11 ± 20.97% in the baseline C group. No differences were observed between the two groups for the other parameters (DT and +dT/dt; Fig 4A and 4B). Increasing [Ca^2+^] from 0.5 to 2.5 mM promoted a greater response in -dT/dt in the C rats than in the obese rats. At calcium concentrations of 1.5 mM, 2.0 mM, and 2.5 mM, the mean percent of -dT/dt was 28.30 ± 13.13%, 29.97 ± 14.90%, and 32.37 ± 16.10%, respectively, of baseline in the Ob group *vs*. 39.03 ± 14.56%, 44.11 ± 18.93%, and 50.08 ± 24.81%, respectively, of baseline in the C group. Therefore, the myocardial dysfunction induced by impaired relaxation in the obese rats was confirmed by the lower response in -dT/dt when compared to the control group.

**Table 2:** Baseline data from isolated muscle preparation.

	Groups	
Variables	C (n = 18)	Ob (n = 17)
**DT (g/mm** ^**2**^ **)**	6.03 ± 1.77	5.30 ± 1.15
**RT (g/mm** ^**2**^ **)**	0.94 ± 0.33	0.88 ± 0.37
**+dT/dt (g/mm** ^**2**^ **/s)**	71.0 ± 21.7	63.9 ± 14.4
**-dT/dt (g/mm** ^**2**^ **/s)**	23.9 ± 5.6	22.5 ± 5.0	
**CSA (mm** ^**2**^ **)**	1.07 ± 0.26	1.21 ± 0.28

Values expressed as mean ± SD. n = number of animals. C: control; Ob: obese; Baseline condition: 2.5 mM [Ca^2+^]. DT: maximum developed tension normalized per cross-sectional area of the papillary muscle; RT: resting tension normalized per cross-sectional area of the papillary muscle; peak of the positive (+dT/dt) and negative (-dT/dt) tension derivatives normalized per cross-sectional area of the papillary muscle; CSA: cross-sectional area. Student’s t-test for independent samples.

**Fig 5:**
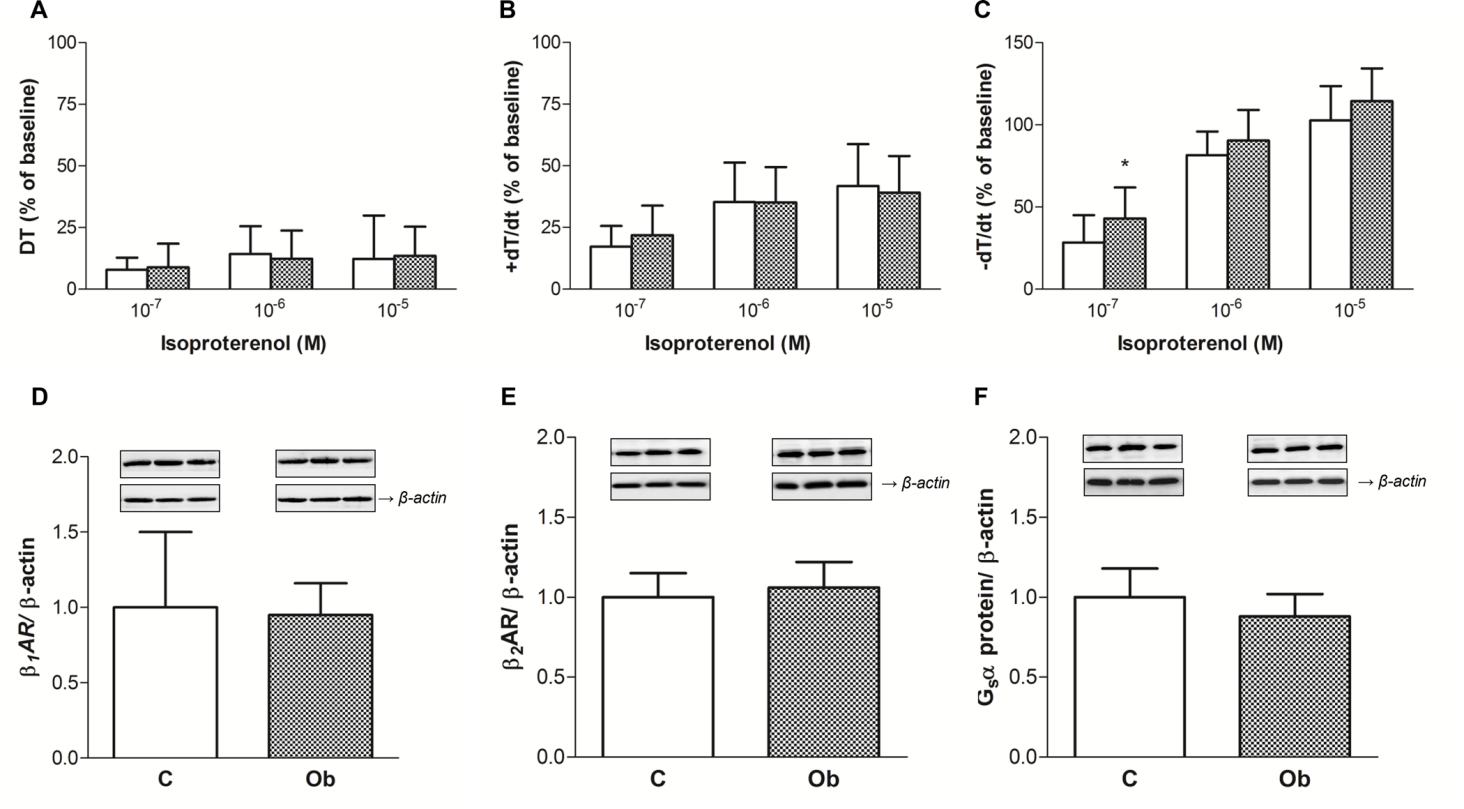
(A, B and C) Effects of increasing isoproterenol concentration in papillary muscles (10^*−7*^ to 10^−5^ M) and (D, E and F) protein expression of β-adrenergic receptors (β-AR) and stimulatory G-protein (G_s_α) from control (white bars) and obese rats (cross-hatched bars). Baseline calcium concentration (1.0 mM) is presented as 100%. Maximum developed tension normalised per cross-sectional area [DT, g/mm^2^] and positive [+dT/dt, g/mm^2^/s] and negative [-dT/dt, g/mm^2^/s] tension derivative normalized per cross-sectional area of the papillary muscle. D: β_1_AR, E: β_2_AR and F: G_s_α. (A, B and C) Data presented as the mean percent of baseline ± standard deviation; Repeated-measures two-way ANOVA and Student-Newman-Keuls *post-hoc* test. (D, E and F) Values shown are mean ± standard deviation; Student’s t-test for independent samples (D, E and F).*p < 0.05 *versus* C.

The effects of *β*-adrenergic stimulation on the papillary muscle function are shown in Fig 5A–5C. For all investigated parameters, the myocardium from the Ob group did not exhibit differences in response to β-adrenoceptor stimulation (isoproterenol) when compared to the C rats (Fig 5A–5C). A minor focal response was observed in -dT/dt in the Ob rats compared with the C rats (Fig 5C). The -dT/dt in the Ob group was greater than in the C group after stimulation by isoproterenol (10^−7^ M) (Fig 5C). At the isoproterenol stimulation of 10^−7^ M, the mean percent of -dT/dt was 43.02 ± 18.89% of baseline in the Ob group *vs*. 28.46 ± 16.56% of baseline in the C group.

The myocardial levels of β-adrenergic receptors (βAR), β_1_AR and β_2_AR, and stimulatory G-protein (G_s_α) were assessed to determine the mechanism for β-adrenergic system-induced changes on cardiac function in the obesity models. Fig 5D–5F show that obesity did not change the protein levels of β_1_AR (C: 1.00 ± 0.5 *vs*. Ob: 0.95 ± 0.21), β_2_AR (C: 1.00 ± 0.15 *vs*. Ob: 1.06 ± 0.16) and G_s_ *α*. (C: 1.00 ± 0.18 *vs*. Ob: 0.88 ± 0.14). Thus, these results indicated that βARs (β_1_AR and β_2_AR) and G_s_α, components of the β-adrenergic system, were not associated with myocardial dysfunction induced by obesity.

## Discussion

The adverse effects of obesity have been extensively studied in experimental models [9,10,49,50]. Interestingly, although several mechanisms have been postulated to identify obesity-induced cardiac dysfunction, little information is available on the relationship between cardiac function and the β-adrenergic system in obesity. The major finding in the current study was that the cardiac dysfunction in the Ob rats induced by unsaturated high-fat diet, after 15 weeks, was not related to β-adrenergic system impairment.

In this study, the unsaturated high-fat diet used was of sufficient intensity and duration to promote obesity in the experimental time period of 15 weeks. According to the literature, fat-enriched diets have been used for decades to model obesity in rodents [9, 10, 51]. The initial moment of obesity occurred in the third week of experimental treatment and the evolution of adiposity remained for more 15 weeks. These results confirm the development of a consistent obesity model in rodents induced by a high unsaturated fat diet. The development of obesity was characterised by significant differences in body weight, fat pads, body fat and adiposity index in the Ob rats compared to the control rats. Although the Ob rats manifested a modest but significant 15.2% increase in total body weight, they developed substantially more adipose tissue than the C rats (97%). Moreover, the adiposity index, an important determinant of obesity, was elevated by 73.3% in the Ob rats compared to the C rats. Authors have reported that diets containing ≥ 30% of energy from fat favours the induction of obesity, demonstrating a positive relationship between levels of dietary fat and the increase in fatty tissue deposits [52–54].

The Ob animal model in this study also presented many disorders that resemble the human comorbidities caused by obesity, such as hypertriglyceridaemia, glucose intolerance, insulin resistance and hyperleptinemia. Furthermore, there was a trend for insulin levels to be greater in obese animals (p = 0.06), indicating the hyperinsulinemia. Consistent with previous investigations, the high-fat diet used in this study was effective at promoting numerous comorbidities associated with short-term obesity [10, 50]. One important aspect of this study is the absence of hypertension in our obesity rodent model. Nascimento et al. [55] evaluated the vascular abnormalities in high-fat diet-induced obesity after 30 weeks. These authors found that the improvement of endothelial relaxation concomitantly increased the bioavailability of nitric oxide (NO), an important vasodilator. The increase of NO synthesis may be a consequence of the hyperinsulinemia and hyperleptinemia observed in obese animals [55]. Therefore, this factor may have been decisive in the absence of hypertension in obesity rodents.

The morphological analysis *post-death* in the current study revealed that obesity induced mild cardiac hypertrophy visualized by increased total heart and left ventricle. This initial process of cardiac remodeling may be regarded as a first step in the sequence of adaptive responses of the heart to stress caused by a large number of physiological and pathological conditions as changes in volume and pressure loads and/or metabolic alterations [56–58]. Rider et al. [59] proposed that cardiac remodeling is an adaptive characteristic of obesity. Thus, obesity-induced changes in cardiac structure may be elicited directly by obesity-induced increases in cardiac loading conditions (preload and afterload) or indirectly by obesity-induced cardiometabolic abnormalities such as dyslipidaemia and insulin resistance/diabetes [60,61]. The literature reports that the insulin resistance induced by obesity with associated hyperinsulinaemia could promote cardiac remodelling via the growth-promoting properties of insulin or by attenuating the anti-apoptotic signalling of the phosphatidylinositol 3'-kinase (PI3K)- Akt (protein kinase B [PKB]) pathway elicited by insulin receptor activation [59,61]. In addition to insulin, the literature also highlights that leptin induces cardiomyocyte hypertrophy in rodents [62]. Thus, hyperleptinemia presented by Ob animals may promote the activation of Ras homolog gene family, member A (RhoA)/Rho-associated coiled-coil-forming protein kinase (ROCK) and p38 mitogen-activated protein kinase (MAPK) protein translocation to the nucleus by its receptor, resulting in cardiomyocyte hypertrophy [63,64]. Our data are in agreement with previous findings that have shown cardiac remodelling in rats with high-fat diet induced by short-term obesity [9,10].

Functional studies performed using isolated papillary muscle allowed us to analyse myocardial function at baseline and after various interventions. The present study showed that obesity after 15 weeks did not change the myocardial function under baseline conditions because all the functional parameters were similar between groups. However, obesity caused damage in-dT/dt after Ca^2+^ stimulation and PRC, altering the relaxation phase. These stimuli provide evidence that the impairment of myocardial function assigned to obesity was related to changes in intracellular Ca^2+^ handling, mainly in the recapture and/or extrusion of cytosolic Ca^2+^ [18]. One explanation for such a result is that the activity and/or levels of sarcoplasmic reticulum (SR) Ca^2+^-ATPase (SERCA2a) may be compromised by obesity; however, previous studies realised in our laboratory demonstrated that SERCA2a is not damaged, but the authors found a reduced phospholamban (PLB) phosphorylated at serine^16^ [36].

Thus, relaxation impairment in obese rats may be related to PLB phosphorylation of Ser^16^ and Thr^17^ by PKA or CaMKII impairment, provided that both are physiologically relevant for controlling SERCA2a activity [65–67]. However, the major stimulus for controlling PLB activity is related to the β adrenergic system, since it PLB phosphorylation at serine^16^ regulates SERCA2a activity, which may hampering or not recapturing Ca^2+^ from sarcoplasmic reticulum. This injury is related to lusitropic damage to cardiac tissue [68]. The transcription signal or signals responsible for triggering the actions of the β-adrenergic system grafting occur because catecholamines or adrenergic agonists bind to their receptors, acting as first messenger in the β-adrenergic pathway. These extracellular stimuli mediated by the action of G_s_α protein binding and the action on adenylate cyclase regulates the concentration of intracellular cAMP (second messenger) in this signalling cascade. The accumulation of the second messenger activation of protein kinase A (PKA) is responsible for the phosphorylation of key proteins in the intracellular Ca^2+^ handling. This cascade of events leads to changes in the activity of proteins including L-type calcium channels [26,69], phospholamban [70], troponin I [71] and ryanodine receptors [72], altering cardiac function [22]. Thus, the adrenergic stimulation increases the inotropy (contractile force), chronotropy (heart rate), dromotropy (excitation conductance), bathmotropy (decrease in threshold of excitation) and lusitropy (relaxation) of cardiac tissue [68].

In the current literature, few studies have evaluated the βA components in experimental models of obesity induced by high-fat diet [34,35,73–76]. Some studies have shown that cardiac function impairment is related to βA system changes [35,73,74], while others researchers have not reported reduced βA response [34,49,75,76]. One explanation for these discrepancies and divergent data existing literature about obesity and βA system may be related to leptin levels, type of diet, animal model utilized and catecholamine levels [49,73–75,77,78]. Meanwhile, it is still unclear whether obesity induced by high-fat diets leads to a reduction in the number of β-adrenergic receptors and/or defects of other components of the β-adrenergic pathway, resulting in cardiac impairment. Carroll et al. [34,35] assessed obese female rabbits for 12 weeks and showed a reduction of cardiac contractile response to β-adrenergic stimulation but no change in affinity and density of receptors. Leopoldo et al. [14] evaluated obese animals fed a high-fat diet for 15 weeks and suggested that the impairment of L-type Ca^2+^ channels is related to β-adrenergic system downregulation. Lima-Leopoldo et al. [36] showed that long-term obesity promotes alterations in diastolic function induced by a reduction of phospholamban phosphorylation at serine^16^. The authors also suggest that the impairment of PLB phosphorylation at serine^16^ in obese rats may be related to βA system downregulation.

Alterations in *β*-adrenergic signalling can occur directly at the receptor level (through altered gene expression or receptor protein concentration) or at the post-receptor level as PKA, adenylyl cyclase activation, cyclic AMP, CaMKII, for example [32,34,79]. However, in disagreement with our hypothesis, the β-adrenergic system did not affect the myocardial function in obesity because there was no difference in the β-adrenergic receptors (β_1_AR and β_2_AR) and stimulatory G-protein (G_s_α). The downregulation activation in obesity could be associated to damage of β-adrenergic signaling into a cascade of biochemical reactions that coordinate cellular responses [20]. The phosphorylation of the receptor is an effective mechanism to modulate the responsiveness of the β-adrenoceptor mediated signal transduction cascade [80]. Thus, the uncoupling of both subtypes of the β receptor could occur due to phosphorylation by PKA, PKC, and by members of the G-protein coupled receptor kinase (GRK) family, also known as βARKs or β-adrenergic receptor kinases [32]. In addition, the downstream intracellular mechanisms involve phosphorylation of numerous substrates by PKA. These targets include regulatory proteins, nuclear transcription factors, ion channels particularly the L-type calcium channel, and metabolic regulatory enzymes as Serca2a [32]. Thus, in the current study, the impairment of myocardial function in the relaxation phase could be related to PLB phosphorylation of Ser^16^ and Thr^17^ by PKA, so post-β receptor components of the β-adrenergic system would be participating. In this context, phospholamban negatively regulates the uptake of calcium by the SR, and a deficiency of PLB phosphorylation can promote impaired left ventricular diastolic performance due to SERCA2a activity damage, resulting in lower recapture and/or extrusion of cytosolic Ca^2+^. Several authors report that cAMP concentration and PKA phosphorylation are mechanisms that are potentially responsible for changes in the myocardial Ca^2+^ handling [17,18,62]. Carroll et al. [81] demonstrated that there was a defect in the cardiac myocyte post-β-receptor signalling pathway in the isolated hearts of obese rabbits. This finding was evidenced by reduced peak pressure +dP/dt, and -dP/dt responses to forskolin stimulation. Another study observed a decrease in PKA activity in *ob/ob* mice, suggesting that the alterations in cardiac performance may be associated with the activity of PLB and/or its phosphorylation, thus altering the Ca^2+^ handling, with consequent myocardial dysfunction [82]. In contrast, Paulino et al. [49] did not find alterations in PKA expression or activity in rats fed a high-fat and high-sucrose diet for 25 weeks.

One alternative explanation for such a result is that the hormonal responsible for modulation of the β-adrenergic system, directly or indirectly, were not impaired by obesity. Previous study has shown the role of leptin on the modulation of the β-adrenergic system [81]. Minhas et al. [82] observed that leptin deficiency, mediated by disruption of signal transduction system, promotes the β-adrenergic depression of myocyte contractility in *ob/ob* mice. The authors observed functional impairment of β-adrenergic response with concomitant decrease in the expression of G_s_α protein; however, changes in the protein expression of the β_1_AR and β_2_AR were not found. The leptin repletion restores depressed β-adrenergic contractility [82]. However, in the current study, the absence of β-adrenergic damage may be due to the hyperleptinemia caused by obesity; no studies have evaluated the resistance to leptin on the β-adrenergic system.

In summary, the myocardial dysfunction caused by obesity, after 15 weeks, was not related to *β*-adrenergic system impairment at the receptor-signalling pathway. Future studies are needed to investigate the influence of obesity induced by an unsaturated high- fat diet on post-β receptor components of the β-adrenergic system and evaluate the catecholamine levels in these obesity models.

## References

[1] Korner J, Arone LJ. The emerging of body weight regulation and its impact on obesity treatment. J Clin Invest. 2003; 111: 565–570. 12618507 10.1172/JCI17953 PMC151906

[2] Brasil. Instituto Brasileiro de Geografia e Estatística—IBGE. Pesquisa de Orçamentos Familiares 2002–2003. Antropometria e análise do estado nutricional de crianças e adolescentes no Brasil. Rio de Janeiro: IBGE; 2006.

[3] Malnick SD, Knobler H. The medical complications of obesity. QJM. 2006; 99: 565–579. 16916862 10.1093/qjmed/hcl085

[4] Poirier P, Giles TD, Bray GA, Hong Y, Stern JS, Pi-Sunyer FX, et al Obesity and cardiovascular disease: pathophysiology, evaluation, and effect of weight loss. Arterioscler Thromb Vasc Biol. 2006; 26: 968–976. 16627822 10.1161/01.ATV.0000216787.85457.f3

[5] Alpert MA. Relation of duration of morbid obesity to left ventricular mass, systolic function, and diastolic filling, and effect of weight loss. Am J Cardiol. 1995; 76: 1194–1197. 7484912 10.1016/s0002-9149(99)80338-5

[6] Scaglione R, Dichiara MA, Indovina A, Lipari R, Ganguzza A, Parrinello G, et al Left ventricular diastolic and systolic function in normotensive obese subjects: influence of degree and duration of obesity. Eur Heart J. 1992; 13: 738–742. 1623860 10.1093/oxfordjournals.eurheartj.a060249

[7] Dong F, Zhang X, Yang X, Esberg LB, Yang H, Zhang Z, et al Impaired cardiac contractile function in ventricular myocytes from leptin-deficient ob/ob obese mice. J Endocrinol. 2006; 188: 25–36. 16394172 10.1677/joe.1.06241

[8] Ren J, Walsh MF, Jefferson L, Natavio M, Ilg KJ, Sowers JR, et al Basal and ethanol-induced cardiac contractile response in lean and obese zucker rat hearts. J Biomed Sci. 2000; 7: 390–400. 10971137 10.1007/BF02255814

[9] Carroll JF, Zenebe WJ, Strange TB. Cardiovascular function in a rat model of diet-induced obesity. Hypertension 2006; 48: 65–72. 16702491 10.1161/01.HYP.0000224147.01024.77

[10] Relling DP, Esberg LB, Fang CX, Johnson WT, Murphy EJ, Carlson EC, et al High-fat diet-induced juvenile obesity leads to cardiomyocyte dysfunction and upregulation of Foxo3a transcription factor independent of lipotoxicity and apoptosis. J Hypertens. 2006; 24: 549–561. 16467659 10.1097/01.hjh.0000203846.34314.94

[11] Fitzgerald SM, Henegar JR, Brands MW, Henegar LK, Hall JE. Cardiovascular and renal responses to a high-fat diet in Osborne-Mendel rats. Am J Physiol Regul Integr Comp Physiol. 2001; 281: R547–R552. 11448859 10.1152/ajpregu.2001.281.2.R547

[12] Carroll JF, Dwyer TM, Grady AW, Reinhart GA, Montani JP, Cockrell K, et al Hypertension, cardiac hypertrophy, and neurohumoral activity in a new animal model of obesity. Am J Physiol. 1996; 271: H373–H378. 8760195 10.1152/ajpheart.1996.271.1.H373

[13] Du toit EF, Nabben M, Lochner A. A potential role for angiotensin II in obesity induced cardiac hypertrophy and ischaemic/reperfusion injury. Basic Res Cardiol. 2005; 100: 346–354. 15821998 10.1007/s00395-005-0528-5

[14] Leopoldo AS, Lima-Leopoldo AP, Sugizaki MM, do Nascimento AF, de Campos DH, Luvizotto RA, et al Involvement of L-type calcium channel and SERCA2A in myocardial dysfunction induced by obesity. J Cell Physiol. 2011; 226: 2934–2942. 10.1002/jcp.22643 21302294

[15] Brainard RE, Watson LJ, Demartino AM, Brittian KR, Readnower RD, Boakye AA, et al High fat feeding in mice is insufficient to induce cardiac dysfunction and does not exacerbate heart failure. PLoS One. 2013; 8: e83174 10.1371/journal.pone.0083174 24367585 PMC3867436

[16] Leopoldo AS, Sugizaki MM, Lima-Leopoldo AP, do Nascimento AF, Luvizotto RA, de Campos DH, et al Cardiac remodeling in a rat model of diet-induced obesity. Can J Cardiol. 2010; 26: 423–429. 20931095 10.1016/s0828-282x(10)70440-2 PMC2954535

[17] Opie LH. Myocardial contraction and relaxation In: Opie LH, editor. The Heart. Physiology from cell to circulation. Philadelphia: Lippincott-Raven; 1998 pp. 209–231.

[18] Bers DM. Cardiac excitation-contraction coupling. Nature 2002; 415: 198–205. 11805843 10.1038/415198a

[19] Lee S, Grafweg S, Schneider T, Jimenez M, Giacobino JP, Ghanem A, et al Total beta-adrenoceptor deficiency results in cardiac hypotrophy and negative inotropy. Physiol Res. 2010; 59: 679–689. 20406048 10.33549/physiolres.931851

[20] Saucerman JJ, Mcculloch AD. Cardiac beta-adrenergic signaling: from subcellular microdomains to heart failure. Ann N Y Acad Sci. 2006; 1080: 348–361. 17132794 10.1196/annals.1380.026

[21] Barros RA, Okoshi MP, Cicogna AC. Beta-adrenergic pathway in healthy and hypertrophied hearts. Arq Bras Cardiol. 1999; 72: 641–656. 10668235 10.1590/s0066-782x1999000500012

[22] Brum PC, Rolim NP, Bacurau AV, Medeiros A. Neurohumoral activation in heart failure: the role of adrenergic receptors. An Acad Bras Cienc. 2006; 78: 485–503. 16936938 10.1590/s0001-37652006000300009

[23] Lymperopoulos A, Bathgate A. Pharmacogenomics of the heptahelical receptor regulators G-protein-coupled receptor kinases and arrestins: the known and the unknown. Pharmacogenomics 2012; 13: 323–341. 10.2217/pgs.11.178 22304582

[24] Brodde OE. Beta-adrenoceptors in cardiac disease. Pharmacol Ther. 1993; 60: 405–430. 7915424 10.1016/0163-7258(93)90030-h

[25] Lymperopoulos A. Physiology and pharmacology of the cardiovascular adrenergic system. Front Physiol. 2013; 4: 240 10.3389/fphys.2013.00240 24027534 PMC3761154

[26] Zhao XL, Gutierrez LM, Chang CF, Hosey MM. The a1-subunit of skeletal muscle L-type Ca channels is the key target for regulation by A-kinase and protein phosphatase-IC. Biochem Biophys Res Commun. 1994; 198: 166–173. 8292020 10.1006/bbrc.1994.1024

[27] Evora PR, Nobre F. The role of G-proteins in the pathophysiology of the cardiovascular diseases. Arq Bras Cardiol. 1999; 72: 209–229. 10488580 10.1590/s0066-782x1999000200009

[28] Adams JW, Brown JH. G-proteins in growth and apoptosis: lessons from the heart. Oncogene 2001; 20: 1626–1634. 11313910 10.1038/sj.onc.1204275

[29] Dincer UD, Bidasee KR, Güner S, Tay A, Ozçelikay AT, Altan VM. The effect of diabetes on expression of beta1-, beta2-, and beta3-adrenoreceptors in rat hearts. Diabetes 2001; 50: 455–461. 11272160 10.2337/diabetes.50.2.455

[30] Moniotte S, Kobzik L, Feron O, Trochu JN, Gauthier C, Balligand JL. Upregulation of beta(3)-adrenoceptors and altered contractile response to inotropic amines in human failing myocardium. Circulation 2001; 103: 1649–1655. 11273992 10.1161/01.cir.103.12.1649

[31] Salazar NC, Chein J, Rockman HA. Cardiac GPCRs: GPCR signaling in healthy and failing hearts. Biochim Biophys Acta. 2007; 1768: 1006–1018. 17376402 10.1016/j.bbamem.2007.02.010 PMC1892229

[32] Lamba S, Abraham WT. Alterations in adrenergic receptor signaling in heart failure. Heart Fai Rev. 2000; 5: 7–16. 10.1023/A:1009885822076 16228912

[33] Fischer V, Gabauer I, Tillinger A, Novakova M, Pechan I, Krizanova O, et al Heart adrenoceptor gene expression and binding sites in the human failing heart. Ann N Y Acad Sci. 2008; 1148: 400–408. 10.1196/annals.1410.013 19120134

[34] Carroll JF, Kyser CK, Martin MM. Beta-Adrenoceptor density and adenylyl cyclase activity in obese rabbit hearts. Int J Obes Relat Metab Disord. 2002; 26: 627–632. 12032745 10.1038/sj.ijo.0801957

[35] Carroll JF, Jones AE, Hester RL, Reinhart GA, Cockrell K, Mizelle HL. Reduced cardiac contractile responsiveness to isoproterenol in obese rabbits. Hypertension 1997; 30: 1376–1381. 9403556 10.1161/01.hyp.30.6.1376

[36] Lima-Leopoldo AP, Leopoldo AS, da Silva DC, do Nascimento AF, de Campos DH, Luvizotto RA, et al Long-term obesity promotes alterations in diastolic function induced by reduction of phospholamban phosphorylation at serine-16 without affecting calcium handling. J Appl Physiol. 2014; 117: 669–678. 10.1152/japplphysiol.00088.2014 24970855 PMC4157165

[37] Surwit RS, Feinglos MN, Rodin J, Sutherland A, Petro AE, Opara EC, et al Differential effects of fat and sucrose on the development of obesity and diabetes in C57BL/6J and A/J mice. Metabolism. 1995; 44: 645–651. 7752914 10.1016/0026-0495(95)90123-x

[38] Levin BE, Richard D, Michel C, Servatius R. Differential stress responsivity in diet-induced obese and resistant rats. Am J Physiol Regul Integr Comp Physiol. 2000; 279: R1357–R1364. 11004005 10.1152/ajpregu.2000.279.4.R1357

[39] Boustany-kari CM, Gong M, Akers WS, Guo Z, Cassis LA. Enhanced vascular contractility and diminished coronary artery flow in rats made hypertensive from diet-induced obesity. Int J Obes. 2007; 31: 1652–1659. 10.1038/sj.ijo.0803426 16819529

[40] Santos PP, Rafacho BP, Gonçalves AF, Jaldin RG, Nascimento TB, Silva MA, et al Vitamin D induces increased systolic arterial pressure via vascular reactivity and mechanical properties. PLoS One. 2014; 9: e98895 10.1371/journal.pone.0098895 24921930 PMC4055656

[41] Ouwens DM, Boer C, Fodor M, de Galan P, Heine RJ, Maassen JA, et al Cardiac dysfunction induced by high-fat diet is associated with altered myocardial insulin signaling in rats. Diabetologia 2005; 48: 1229–1237. 15864533 10.1007/s00125-005-1755-x

[42] Pitombo C, Araújo EP, Souza CT, Pareja JC, Geloneze B. Amelioration of diet-induced diabetes mellitus by removal of visceral fat. J Endocrinol. 2006; 191: 699–706. 17170226 10.1677/joe.1.07069

[43] Matthews DR, Hosker JP, Rudenski AS, Naylor BA, Treacher DF, Turner RC. Homeostasis model assessment: insulin resistance and beta-cell function from fasting plasma glucose and insulin concentrations in man. Diabetologia. 1985; 28: 412–419. 3899825 10.1007/BF00280883

[44] Fioresi M, Simões MR, Furieri LB, Broseghini-Filho GB, Vescovi MV, Stefanon I, et al Chronic lead exposure increases blood pressure and myocardial contractility in rats. PLoS One. 2014; 9: e96900 10.1371/journal.pone.0096900 24841481 PMC4026242

[45] Riou B, Lecarpentier Y, Viars P. Inotropic effect of ketamine on rat cardiac papillary muscle. Anesthesiology 1989; 1: 116–125. 10.1097/00000542-198907000-00020 2751123

[46] David JS, Vivien B, Lecarpentier Y, Coriat P, Riou B. Interaction of protamine with alpha- and beta-adrenoceptor stimulations in rat myocardium. Anesthesiology 2002; 96: 521. 10.1097/00000542-200111000-00029 11684994

[47] Hanouz JL, Riou B, Massias L, Lecarpentier Y, Coriat P. Interaction of halothane with alpha- and beta-adrenoceptor stimulations in rat myocardium. Anesthesiology 1997; 86: 147–159. 9009950 10.1097/00000542-199701000-00019

[48] Porter KE, Turner NA. Cardiac fibroblasts: at the heart of myocardial remodeling. Pharmacol Ther. 2009; 123: 255–278. 10.1016/j.pharmthera.2009.05.002 19460403

[49] Paulino EC, Ferreira JC, Bechara LR, Tsutsui JM, Mathias W Jr, Lima FB, et al Exercise training and caloric restriction prevent reduction in cardiac Ca^2+^ handling protein profile in obese rats. Hypertension 2010; 56: 629–635. 10.1161/HYPERTENSIONAHA.110.156141 20644006

[50] Ren J, Zhu BH, Relling DP, Esberg LB, Ceylan-Isik AF. High-fat diet-induced obesity leads to resistance to leptin-induced cardiomyocyte contractile response. Obesity 2008; 16: 2417–2423. 10.1038/oby.2008.381 18719678

[51] Buettner R, Parhofer KG, Woenckhaus M, Wrede CE, Kunz-Schughart LA, Schölmerich J, et al Defining high-fat-diet rat models: metabolic and molecular effects of different fat types. J Mol Endocrinol. 2006; 36: 485–501. 16720718 10.1677/jme.1.01909

[52] Lima-Leopoldo AP, Leopoldo AS, Silva DC, Nascimento AF, Campos DH, Luvizotto RA, et al Influence of long-term obesity on myocardial gene expression. Arq Bras Cardiol. 2013; 100: 229–237. 23598576 10.5935/abc.20130045

[53] White PA, Cercato LM, Araújo JM, Souza LA, Soares AF, Barbosa AP, et al Model of high-fat diet-induced obesity associated to insulin resistance and glucose intolerance. Arq Bras Endocrinol Metab. 2013; 57: 339–345. 10.1590/s0004-27302013000500002 23896799

[54] Hariri N, Thibault L. High-fat diet-induced obesity in animal model. Nutr Res Rev. 2010; 23: 270–299. 10.1017/S0954422410000168 20977819

[55] Nascimento TB, Baptista RF, Pereira PC, Campos DH, Leopoldo AS, Leopoldo AP, et al Vascular alterations in high-fat diet-obese rats: role of endothelial L-arginine/NO pathway. Arq Bras Cardiol. 2011; 97: 40–45. 21603776 10.1590/s0066-782x2011005000063

[56] Skurk C, Izumiya Y, Maatz H, Razeghi P, Shiojima I, Sandri M, et al The FOXO3a transcription factor regulates cardiac myocyte size downstream of AKT signaling. J Biol Chem. 2005; 280: 20814–20823. 15781459 10.1074/jbc.M500528200 PMC3632436

[57] Abel ED, Litwin SE, Sweeney G. Cardiac remodeling in obesity. Physiol Rev. 2008; 88: 389–419. 10.1152/physrev.00017.2007 18391168 PMC2915933

[58] Frey N, Katus HA, Olson EN, Hill JA. Hypertrophy of the heart: a new therapeutic target? Circulation. 2004; 109: 1580–1589. 15066961 10.1161/01.CIR.0000120390.68287.BB

[59] Rider OJ, Francis JM, Ali MK, Byrne J, Clarke K, Neubauer S, et al Determinants of left ventricular mass in obesity; a cardiovascular magnetic resonance study. J Cardiovasc Magn Reson. 2009; 11: 9 10.1186/1532-429X-11-9 19393079 PMC2680851

[60] Wensley I, Salaveria K, Bulmer AC, Donner DG, du Toit EF. Myocardial Structure, Function, and Ischaemic Tolerance in a Rodent Model of Obesity with Insulin Resistance. Exp Physiol. 2013; 98: 1552–1564. 10.1113/expphysiol.2013.074948 23851919

[61] Dhanasekaran A, Gruenloh SK, Buonaccorsi JN, Zhang R, Gross GJ, Falck JR, et al Multiple antiapoptotic targets of the PI3K/Akt survival pathway are activated by epoxyeicosatrienoic acids to protect cardiomyocytes from hypoxia/anoxia. Am J Physiol Heart Circ Physiol. 2008; 294: H724–H735. 18055514 10.1152/ajpheart.00979.2007 PMC2443685

[62] Leifheit-Nestler M, Wagner NM, Gogiraju R, Didié M, Konstantinides S, Hasenfuss G, et al Importance of leptin signaling and signal transducer and activator of transcription-3 activation in mediating the cardiac hypertrophy associated with obesity. J Trans Med. 2013; 11: 170. 10.1186/1479-5876-11-170 PMC3717024 23841921

[63] Zeidan A, Javadov S, Chakrabarti S and Karmazyn M. Leptin-induced cardiomyocyte hypertrophy involves selective caveolae and RhoA/ROCK-dependent p38 MAPK translocation to nuclei. Circ Rev. 2008; 77: 64–72. 10.1093/cvr/cvm020 18006472

[64] Zeidan A, Javadov S, Chakrabarti S, Karmazyn M. mTOR mediated RhoA/ROCK-dependent leptin-induced cardiomyocyte hypertrophy. Mol Cell Biochem. 2011; 352: 99–108. 10.1007/s11010-011-0744-2 21318349

[65] Ablorh NA, Miller T, Nitu F, Gruber SJ, Karim C, Thomas DD. Accurate quantitation of phospholamban phosphorylation by immunoblot. Anal Biochem. 2012; 425: 68–75. 10.1016/j.ab.2012.01.028 22369895 PMC3338889

[66] Luo W, Chu G, Sato Y, Zhou Z, Kadambi VJ, Kranias EG. Transgenic approaches to define the functional role of dual site phospholamban phosphorylation. J Biol Chem. 1998; 273: 4734–4739. 9468536 10.1074/jbc.273.8.4734

[67] Sayadi M, Feig M. Role of conformational sampling of Ser16 and Thr17-phosphorylated phospholamban in interactions with SERCA. Biochim Biophys Acta. 2013; 1828: 577–585. 10.1016/j.bbamem.2012.08.017 22959711

[68] Bögeholz N, Muszynski A, Pott C. The physiology of cardiac calcium handling. Wien Med Wochenschr. 2012; 162: 278–282. 22707075 10.1007/s10354-012-0102-3

[69] Gerhardstein BL, Puri TS, Chien AJ, Hosey MM. Identification of the sites phosphorylated by cyclic AMP-dependent protein kinase on the beta 2 subunit of L-type voltage-dependent calcium channels. Biochemistry 1999; 38: 10361–10370. 10441130 10.1021/bi990896o

[70] Simmerman HK, Jones LR. Phospholamban: protein structure, mechanism of action and role in cardiac function. Physiol Rev. 1998; 78: 921–947. 9790566 10.1152/physrev.1998.78.4.921

[71] Sulakhe PV, Vo XT. Regulation of phospholamban and troponin-I phosphorylation in the intact rat cardiomyocytes by adrenergic and cholinergic stimuli. Mol Cell Biochem. 1995; 149–150: 103–126. 8569720 10.1007/BF01076569

[72] Marx SO, Reiken S, Hisamatsu Y, Jayaraman T, Burkhoff D, Rosemblit N, et al PKA phosphorylation dissociates FKBPI2.6 from the calcium release channel (ryanodine receptor) defective regulation in failing hearts. Cell 2000; 101: 365–376. 10830164 10.1016/s0092-8674(00)80847-8

[73] Dincer UD. Cardiac β-adrenoreceptor expression is markedly depressed in Ossabaw swine model of cardiometabolic risk. Int J Gen Med. 2011; 4: 493–499. 10.2147/IJGM.S18175 21760751 PMC3133518

[74] Cabrol P, Galinier M, Fourcade J, Verwaerde P, Massabuau P, Tran MA, et al Functional decoupling of left ventricular beta-adrenorecptor in a canine model of obesty-hypertension. Arch Mal Coeur Vaiss. 1998; 91: 1021–1024. 9749157

[75] Pinotti MF, Silva MD, Sugizaki MM, Novelli YS, Sant'ana LS, Aragon FF, et al Influences of rich in saturated and unsaturated fatty acids diets in rat myocardium. Arq Bras Cardiol. 2007; 88: 346–353. 17533478 10.1590/s0066-782x2007000300015

[76] Lima-Leopoldo AP, Leopoldo AS, Sugizaki MM, Bruno A, Nascimento AF, Luvizotto RA, et al Myocardial dysfunction and abnormalities in intracellular calcium handling in obese rats. Arq Bras Cardiol 2011; 97: 232–240. 21584481 10.1590/s0066-782x2011005000061

[77] Ponsard B, Durot I, Fournier A, Oudot F, Athias P, Grynberg A. Long-chain polyunsaturated fatty acids influence both β- and α-adrenergic function of rat cardiomyocytes. JAOCS 1998; 75: 247–254.

[78] Illiano G, Naviglio S, Pagano M, Spina A, Chiosi E, Barbieri M, Paolisso G. Leptin affects adenylate cyclase activity in H9c2 cardiac cell line: effects of short- and long-term exposure. Am J Hypertens. 2002; 15: 638–643. 12118913 10.1016/s0895-7061(02)02925-4

[79] Grimm M, Brown JH. Beta-adrenergic receptor signaling in the heart: role of CaMKII J Mol Cell Cardiol. 2010; 48: 322–330. 10.1016/j.yjmcc.2009.10.016 19883653 PMC2896283

[80] Wallukat G. The β-Adrenergic Receptors. Herz 2002; 27:683–690. 12439640 10.1007/s00059-002-2434-z

[81] Carroll JF. Post-beta-receptor defect in isolated hearts of obese-hypertensive rabbits. Int J Obes Relat Metab Disord. 1999; 23: 863–866. 10490788 10.1038/sj.ijo.0800964

[82] Minhas MK, Khan SA, Raju SV, Phan AC, Gonzalez DR, Skaf MW, et al Leptin repletion restores depressed *β*-adrenergic contractility in *ob/ob* mice independently of cardiac hypertrophy. J Physiol. 2005; 565: 463–474. 15760936 10.1113/jphysiol.2005.084566 PMC1464532

